# Supplementary material for: Causal Relationship between Aging and Anorexia Nervosa: A White-Matter-Microstructure-Mediated Mendelian Randomization Analysis
Source: Biomedicines. 2024 Aug 16;12(8):1874. doi: 10.3390/biomedicines12081874 (PMC11351342; doi:10.3390/biomedicines12081874)
Supplement: Supplementary file 1 [file biomedicines-12-01874-s001.zip › Supplementary Figure/Figure S4/Singe SNP plot of MR analysis for TL to ubm-b-1747.pdf]

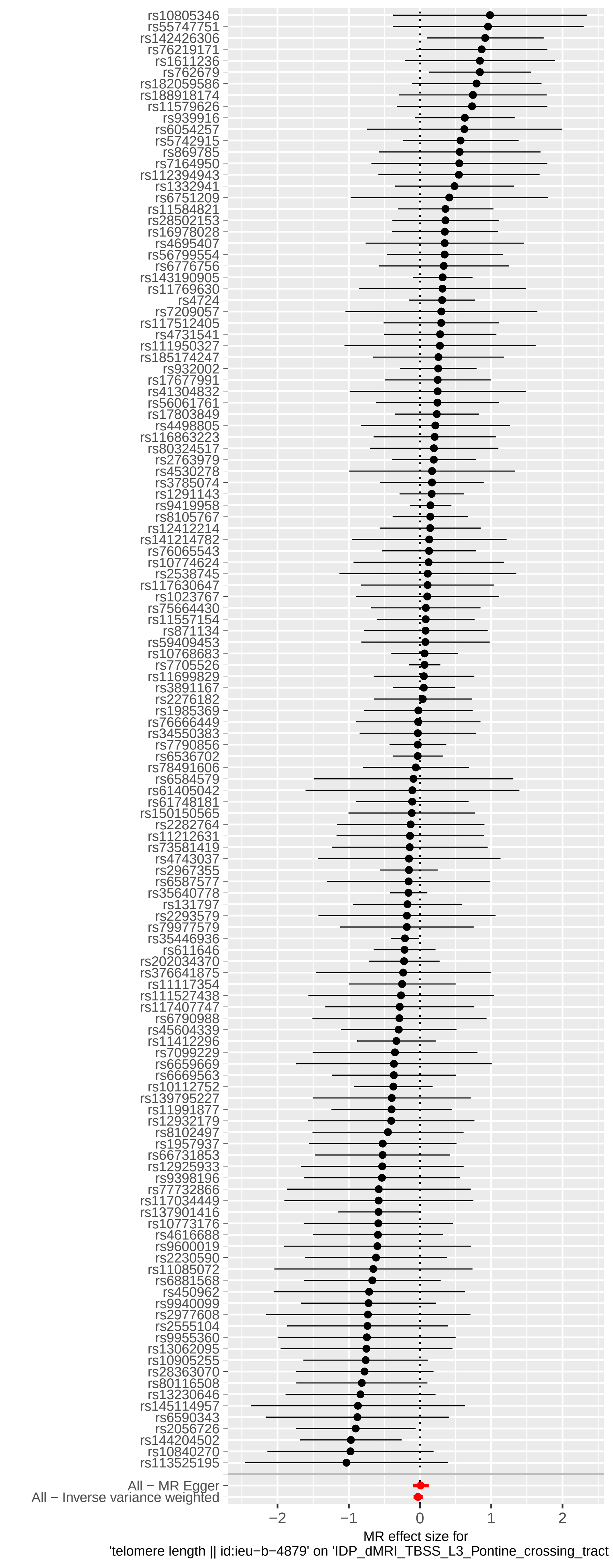
rs10805346
rs55747751
rs142426306
rs76219171
rs1611236
rs762679
rs182059586
rs188918174
rs11579626
rs939916
rs6054257
rs5742915
rs869785
rs7164950
rs112394943
rs1332941
rs6751209
rs11584821
rs28502153
rs16978028
rs4695407
rs56799554
rs6776756
rs143190905
rs11769630
rs4724
rs7209057
rs117512405
rs4731541
rs111950327
rs185174247
rs932002
rs17677991
rs41304832
rs56061761
rs17803849
rs4498805
rs116863223
rs80324517
rs2763979
rs4530278
rs3785074
rs1291143
rs9419958
rs8105767
rs12412214
rs141214782
rs76065543
rs10774624
rs2538745
rs117630647
rs1023767
rs75664430
rs11557154
rs871134
rs59409453
rs10768683
rs7705526
rs11699829
rs3891167
rs2276182
rs1985369
rs76666449
rs34550383
rs7790856
rs6536702
rs78491606
rs6584579
rs61405042
rs61748181
rs150150565
rs2282764
rs11212631
rs73581419
rs4743037
rs2967355
rs6587577
rs35640778
rs131797
rs2293579
rs79977579
rs35446936
rs611646
rs202034370
rs376641875
rs11117354
rs111527438
rs117407747
rs6790988
rs45604339
rs11412296
rs7099229
rs6659669
rs6669563
rs10112752
rs139795227
rs11991877
rs12932179
rs8102497
rs1957937
rs66731853
rs12925933
rs9398196
rs77732866
rs117034449
rs137901416
rs10773176
rs4616688
rs9600019
rs2230590
rs11085072
rs6881568
rs450962
rs9940099
rs2977608
rs2555104
rs9955360
rs13062095
rs10905255
rs28363070
rs80116508
rs13230646
rs145114957
rs6590343
rs2056726
rs144204502
rs10840270
rs113525195
All − MR Egger
All − Inverse variance weighted
−2
−1
0
1
2
MR effect size for
'telomere length || id:ieu−b−4879' on 'IDP_dMRI_TBSS_L3_Pontine_crossing_tract
